# Supplementary material for: Learning curve and surgical outcome of robotic assisted colorectal surgery with ERAS program
Source: Sci Rep. 2022 Nov 29;12:20566. doi: 10.1038/s41598-022-24665-w (PMC9709162; doi:10.1038/s41598-022-24665-w)
Supplement: Supplementary file 2 — Supplementary Information 2. [file 41598_2022_24665_MOESM2_ESM.zip › RAL Raw data-Ñ[▒K/105/OP N_ñw╝╨░O▒Kñσ.pdf]

W92 / W92

臺中榮民總醫院
手術室護理記錄

[redacted]

基本資料

- 室別： 開刀房-17室，手術科別： CRS，手術類別： 常規，日期： 2021/08/18
- 進入時間： 0805 ； 麻醉開始時間： 0820 ； Time Out時間： 0915 ； 劃刀時間： 0916 ； 手術結束時間： 1305 送出時間： 1315 ；
- 麻醉方式： GE
  診斷：appendix cancer.
  手術名稱：Robot Assisted Colectomy(R)
  手術局部用藥：nil
  術前預防性抗生素：
  - 抗生素名稱一： Veterin 1GM IVD/由麻醉護理人員給予
  - 時間： 0900
  - 上次簽章: 歐真勤

  ，追加預防性抗生素：
- 手術醫師： 林俊余，楊曙榮，洪浚嚴
- 麻醉醫師： VS 賴慧卿
-
  - 刷手護士A ： 廖子晴，時間： 0805，刷手護士B ： 陳家瑋，時間： 0805，接替刷手一： 張秀敏，時間： 1200，接替刷手二： 曹薇，時間： 1200
  - 流動護士A ： 歐真勤，時間： 0805，接替流動一： 陳家瑋，時間： 1200

皮膚狀況

A-V Shunt： 無
術前： 完整
記錄者： 上次簽章: 歐真勤
術後： 不完整
部位(術後)：

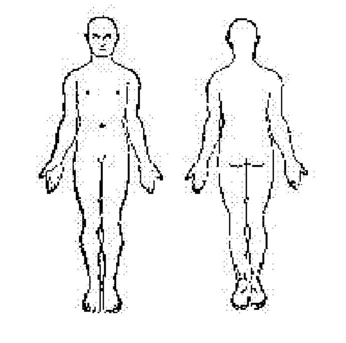

右上臂因壓脈帶造成線性皮下出血

記錄者： 陳家瑋

手術準備

手術臥式 單一手術臥式

-
  - 手術臥式： 仰臥
  - 使用附件： 溫毯 38 ℃，頭圈
  - 皮膚準備： Hibiscrub，2% Chlorhexidine
  - 電燒： 使用電燒: 電極片位置： 其它： 導電軟墊，雙極電燒，記錄者： 上次簽章: 歐真勤

止血帶T： 無

植入物

- 植入物名稱/號碼/數量/部位：GIA:45-4.3mmX2/colon

, 記錄者： 上次簽章: 歐真勤

標本送檢

- 細菌培養 無
- 細胞學 無
- 冰凍切片 無
- 病理標本
  組織名稱一： A-colon
  ,
  組織名稱二： Ileocolic root
- 截肢 無

標本交付 標本： 1 袋，標本： 0 盒，標本： 1 瓶，細胞學： 0 管，Culture Tube： 0 管，被交付醫師： Fellow 楊曙榮，

- 記錄者： 上次簽章: 陳家瑋
- 時間： 1245

傷口術後敷料

傷口敷料： 傷口敷料： 無

- 請於圖上標示部位：

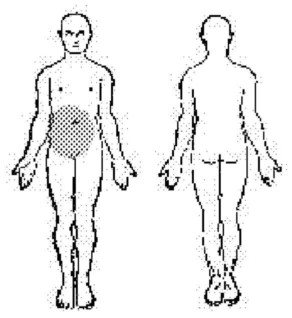
腹部trocar洞

- 傷口敷料： mepore

導管

導管	天數	導管資訊	插入日期	插入時間	更換日期	拔除日期	拔除時間	拔除原因	預計拔除/更換	預計拔除/更換日期
Foley(1)	1	號碼:16 Fr.; 部位:膀胱;	2021/08/18	0840						
Penrose Drain(1)	1	號碼:12 mm.; 部位:肝下;	2021/08/18	1215						
Epidural PCA(1)	1	部位:硬脊膜;	2021/08/18	0830						
A-line (1)	1	號碼:20 Ga; 部位:右手;	2021/08/18	0845						

用物計數

- 手術中出血量： 100 c.c.
- 手術中尿量： 300 c.c.
-
  - 其它： 無

用物計數： 多切口同時縫合

- 1.器械： 正確，2.縫針： 正確，3.刀片： 正確，4.紗布： 正確，5.腹部墊： 未使用，6.紗球： 未使用，7.PEANUTS ： 未使用，8.紗條： 正確，9.絲帶： 未使用，10.針頭： 未使用，11.Vessel loop： 未使用，12.Boots： 未使用，13.Jelco： 未使用，14.棉條： 未使用，
  - 第一次：
    - 刷手護士 ： 張秀敏
    - 流動護士： 上次簽章: 陳家瑋
    - 時間： 1225

  ,
  - 第二次：

- 刷手護士 ： 張秀敏
- 流動護士： 上次簽章: 陳家瑋
- 時間： 1230

,

- 第三次：
  - 刷手護士 ： 張秀敏
  - 流動護士： 上次簽章: 陳家瑋
  - 時間： 1235

備註

備註： davinci0scopy:NO2

- 記錄者： 上次簽章: 歐真勤
- 時間： 0805

填寫護理人員 陳家瑋

正式摘要

W92 / W92

doctorRank:B
